# Hybrid Path Planning for Efficient Data Collection in UAV-Aided WSNs for Emergency Applications

**DOI:** 10.3390/s21082839

**Published:** 2021-04-17

**Authors:** Sabitri Poudel, Sangman Moh

**Affiliations:** Department of Computer Engineering, Chosun University, 309 Pilmun-daero, Dong-gu, Gwangju 61452, Korea; poudelsabitri@chosun.kr

**Keywords:** wireless sensor network, data gathering, unmanned aerial vehicle, path planning, artificial bee colony, collision avoidance, delay minimization, probabilistic roadmap

## Abstract

In unmanned aerial vehicle (UAV)-aided wireless sensor networks (UWSNs), a UAV is employed as a mobile sink to gather data from sensor nodes. Incorporating UAV helps prolong the network lifetime and avoid the energy-hole problem faced by sensor networks. In emergency applications, timely data collection from sensor nodes and transferal of the data to the base station (BS) is a prime requisite. The timely and safe path of UAV is one of the fundamental premises for effective UWSN operations. It is essential and challenging to identify a suitable path in an environment comprising various obstacles and to ensure that the path can efficiently reach the target point. This paper proposes a hybrid path planning (HPP) algorithm for efficient data collection by assuring the shortest collision-free path for UAV in emergency environments. In the proposed HPP scheme, the probabilistic roadmap (PRM) algorithm is used to design the shortest trajectory map and the optimized artificial bee colony (ABC) algorithm to improve different path constraints in a three-dimensional environment. Our simulation results show that the proposed HPP outperforms the PRM and conventional ABC schemes significantly in terms of flight time, energy consumption, convergence time, and flight path.

## 1. Introduction

In recent years, unmanned aerial vehicles (UAVs) have been used increasingly in many fields. UAVs can accomplish extremely difficult or dangerous tasks for human beings, such as firefighting, disaster relief, and search and rescue [1,2,3]. Owing to UAVs’ ability to sense, monitor, and maneuver on a broader area within a short period of time, their use is becoming more popular in many applications [4,5,6,7,8]. UAVs have also played very significant roles in the face of the global COVID-19 pandemic. UAVs are used as a means of transportation to pick up and deliver test samples and medical supplies to minimize the risk of exposure and time. In many countries, UAVs are employed for the aerial spraying of disinfectant in potentially contaminated places.

Moreover, UAVs are suitable for use in wireless sensor networks (WSNs) as mobile sinks to collect data from sensors powered by limited energy and then transfer them to the base station (BS) [9,10]. Typically, UAVs are incorporated in WSNs to minimize the energy consumption of sensor nodes. In most WSN applications, sensor nodes are deployed in areas where replacing or recharging the batteries of small and energy-constrained sensors is challenging or impossible. In our study, we used a UAV as a mobile data collector in WSNs in emergency environments. The emergency environments indicate post-war and post-disaster scenarios that lack a fully connected network of sensor nodes (SNs) [11,12]. We cannot rely solely on arrangements built previously, as the established network might not remain or function [13]. The use of UAVs to support SNs is promising for infrastructure-less integration. Hence, UAV-aided WSNs (UWSNs) are investigated in this study to reduce the energy consumption of sensors [14] and minimize the network latency.

In a dynamic environment, there may be obstacles to the accomplishment of different UAV missions. Thus, UAVs must have the autonomy to perform various operations using sensors, a global positioning system (GPS), and cameras [15,16]. Path planning in UWSNs differs from path planning in pure UAV networks. A routing algorithm for beamforming-constrained swarm networking to improve the network capacity is studied in [17]. In [18], a distributed system to identify the appearance and the approximate position of unwelcome drones using a wireless acoustic sensor network and machine learning algorithms is studied. A lightweight blockchain-based secure routing algorithm for swarm UAV networking is proposed in [19]. Path planning in UWSNs aims to identify the optimal flight path from the starting point to the target point by considering the flying time, threats, and optimum height and velocity for efficient data-gathering from SNs [20]. Many path planning methods can be used for UAVs to navigate in a three-dimensional (3D) domain filled with obstacles.

Path planning can be achieved using classical or heuristic algorithms. Classical algorithms are simple and can be implemented easily; however, they may not efficiently yield complex and dynamic environments. These include cell decomposition, potential field, and sampling-based methods. Probabilistic roadmap (PRM) and rapidly exploring random tree (RRT) schemes belong to classical algorithms. Heuristic algorithms are used for optimization and robustness. They include neural network, fuzzy logic, and nature-inspired algorithms. Genetic algorithms (GA), particle swarm optimization (PSO), ant colony optimization (ACO), and artificial bee colony (ABC) schemes belong to heuristic algorithms. Our study has exploited a hybrid approach involving both classical and heuristic algorithms to achieve better results.

### 1.1. Motivation

**Figure 1:**
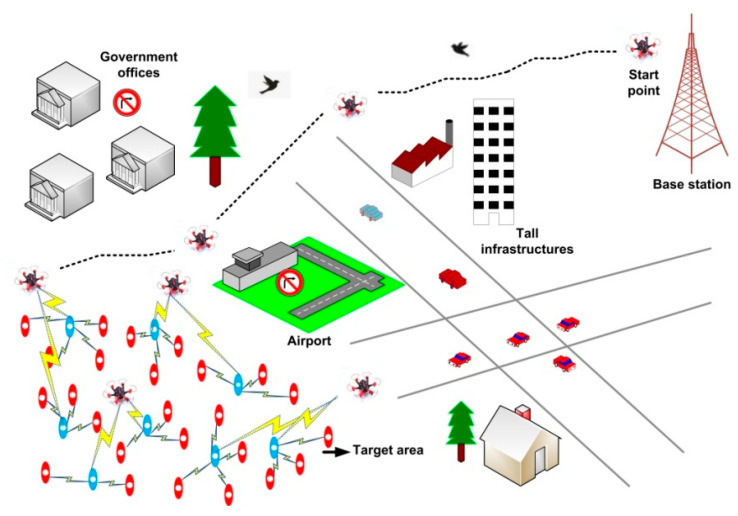
Example of UAV path planning in a UWSN.

We have targeted time-critical and emergency environments in our work. In such scenarios, data delivery must be performed in a timely manner, such that timely actions can be performed; otherwise, lives and properties may be lost [21,22]. UWSNs for emergency applications must operate in harsh environments, where data collection must be performed in dynamic environments with high reliability while ensuring quick responses [23]. To achieve better network performances in such environments, several issues pertaining to UWSNs must be addressed [24]. Path generation is a complicated procedure owing to various operating constraints such as waypoint covering, collision detection and avoidance, and flight-time reduction. Identifying the safest and shortest path that ensures minimal energy consumption and delay is critical in the path planning of UWSNs in emergency environments. UAVs’ path planning for self-directed exploration is a promising topic that has garnered significant attention [25]. However, we do not find any path planning algorithms in the literature that deal with emergencies’ constraints and provide the shortest and safest path for efficient data collection from the ground sensors. UAV path planning in a 3D dynamic environment with static/dynamic obstacles and prohibited areas is challenging and not much studied. Hence, in this study, we have focused on path planning that involves traversing an emergency environment without colliding with environmental elements to achieve the predefined goal. Figure 1 shows an example of UAV path planning in a UWSN.

### 1.2. Contributions and Organization of the Paper

The proposed hybrid algorithm aims to navigate the most acceptable flight route in a complex environment without colliding with environmental elements by combining the PRM [26] and ABC [27] algorithms. Besides this, optimization of the different environments is considered to achieve the best possible path. PRM and ABC’s hybridization and optimization have not yet been tested to the best of our knowledge. However, a hybrid of PRM and ABC provides a better convergence rate and accuracy than other algorithms. In the proposed HPP, the PRM roadmap is used as an input by the ABC algorithm, where a swarm of bees (particles) coordinates with different environmental constraints to obtain the shortest and safest path.

The key contributions of our study are as follows:

- We incorporate a UAV as a communication network facilitator for efficient data gathering from WSNs in emergency environments. The UAV uses directional antennas and comprises two wireless transceivers operating concurrently for timely and energy-efficient UAV to cluster head (CH) communication;
- The proposed model uses an energy-efficient clustering technique which plays a significant role in reducing delay and energy of UWSNs;
- The proposed hybrid path planning algorithm uses the PRM to obtain the shortest and collision-free roadmap, followed by the modified ABC, to dynamically find the position coordinates if some threats or obstacles arise during the flight time;
- The proposed HPP algorithm enhances the wake-up schedule of CHs in the target WSN with the UAV’s path to minimize the energy consumption of the CHs, ensuring that data are collected efficiently and fairly from the ground sensors;
- The UAV path is optimized to avoid static and dynamic obstacles, prohibited areas, and different environmental constraints (such as wind speed, atmospheric irregularities, and wind pressure). The UAV’s height and velocity are optimized to minimize the UAV flight time and the sensors’ energy consumption. The turning angle of UAV is also considered to obtain a smoother path;
- Our performance study based on MATLAB simulations shows that the proposed hybrid path planning algorithm outperforms the conventional algorithms in terms of flight time, energy usage, accuracy, and safety. The convergence time of the proposed algorithm is also compared with some other popular path planning algorithms.

The remainder of this paper is organized as follows. The existing UAV path planning techniques are reviewed in the following section. In Section 3, two typical UAV path planning algorithms are investigated, and a network model is presented. In Section 4, the proposed hybrid path planning algorithm is presented with different optimization constraints. In Section 5, the proposed algorithm’s performance is evaluated via computer simulation and compared with the PRM and ABC algorithms. Finally, the conclusions and future works are presented in Section 6.

## 2. Related Works

In recent years, UWSN applications have attracted considerable attention from industry and academia. UAV flight path optimization has been considered in several prior studies [28,29] from different perspectives related to the targeted application. A range of techniques related to mobile robots in WSNs is reviewed in [30]. Additionally, an extensive survey on recently developed methods that exploit the motion of sensor nodes and/or sink(s) to prolong the lifetime of mobile WSNs is presented in [31]. Each study aims to optimize specific parameters, such as the consumed energy, flight duration, travel distance, and task offload efficiency.

In [32], ACO-based mobile sink path determination for wireless sensor networks was proposed to maximize the network lifetime and minimize the delay in collecting data from the sensor nodes. UAV path planning with safety requirements was primarily investigated. In [33], the collision-free trajectory-planning algorithm based on RRT and optimized RRT (RRT*) was studied for solving detected conflicts for UWSNs.

A new path planning algorithm based on spiral decomposition, called the spiral path planning algorithm, was proposed for WSNs deployed in remote tactical edge networks that did not have the required infrastructure [34]. The optimal trajectory planning for UAV-assisted data collection in WSNs under the age of data constraints was elaborated in [35]. In [36], a PSO-based optimization method was proposed to obtain waypoints for a UAV to reduce the energy consumption and bit error rate of SNs. The UAV travel time was presented. The cooperative relay was employed for efficient data gathering, and waypoints were selected freely. An algorithm named efficient routing strategy for UAV, based on ACO for UWSNs, was proposed to monitor farmland information in remote mountain areas [37]. In [38], a simulated annealing algorithm was employed to detect the optimal flight trajectory based on the WSN architecture. In [39], deep learning (DL) trained by a GA was presented to improve path planning efficiency for data collection using multiple UAVs. 6 G and deep reinforcement learning (DRL)-based path planning is studied in [40]. Joint genetic algorithm and ant colony optimization for optimal path selection in accordance with sensing, energy, time, and risk utilities are discussed in [41]. Dynamic programming (DP)-based path planning is studied for path planning in UWSNs in [42]. The existing works for path planning in UWSNs are summarized in Table 1.

From the literature study, we can say that most of the path planning works in the literature are dedicated to finding a collision-free, energy-efficient, and age-optimal path. They all contribute to an effective path planning approach. However, they do not address 3D network scenarios and the different constraints of dynamic environments. Moreover, for emergency environments, a delay-sensitive algorithm is highly desired. Hence, we have attempted to design the shortest and safest path between the target area and the BS in a 3D dynamic environment using the shortest roadmap and the fastest converging optimized ABC algorithm.

## 3. Formulation

In this study, two typical UAV path planning algorithms are investigated, namely the PRM and ABC algorithms. Subsequently, the system model pertaining to our study is explicitly formulated.

Table 2 shows the symbols and notations used herein, along with their definitions.

### 3.1. PRM

Among the different probabilistic sampling-based algorithms, the PRM [27] is an extremely effective path planning approach due to its probabilistic completeness and remarkable practical performance. The PRM is completed in two steps, i.e., the learning and query phases. In the learning phase, the roadmap of the application environment is constructed in a probabilistic manner. It is represented as an undirected graph *G = {V, E*}, where *V* is the set of configurations of UAV in free space, and *E* correspond to the path connecting the nodes in the configurations.

In the query phase, the constructed roadmap is used to solve path planning issues. Primarily, the graph *G = {V, E*} is empty. Several random configurations are generated in the free space and added to *V*. At the starting and ending positions of the UAV, i.e., *P_start_* and *P_end_*, respectively, nodes from *V* are verified for collision and possible connections and then joined to obtain a feasible path. Let *γ* be the set of shortest paths between pairs of nodes in *V*, and $n^{\prime }$ be the number of total path samples required to obtain the targeted path. Therefore, the shortest path generated by the PRM is expressed as

(1) $$P_{shortest}=\overset{n}{\underset{i=1}{\sum }}\gamma \times P_{l},$$

where *P_l_* is the path length. To minimize the number of failures during the selection of nodes from *V*, a new set $N_{P_{start}}$ is defined that maintains the closest neighbors of *P_start_*; it is expressed as

(2) $$N_{P_{start}}=\left\{R\epsilon V|D\left(R,R^{\prime }\right)\leq D_{max}\right\},$$

where *R* is the nearest neighbor of *P_start_*, *R’* the nearest neighbor of *R*, and *D_max_* the maximum distance. *D (R, R’*) is the Euclidean distance between *R* and *R*’, expressed as

(3) $$D\left(R,\;R^{\prime }\right)=\left|UAV_{\left(R^{\prime }\right)}-UAV_{\left(R\right)}\right|.$$

During the expansion step, ω(R), the measure of path complexity of the coordinate *R*, is used. If the value of ω(R) is greater than the previous coordinate, then the coordinate in *R* is avoided. Subsequently, a new node from *V* is selected using the probabilistic scheme, as follows

(4) Prob(R)=ω(R),

with ω(R) expressed as

(5) $$\omega \left(R\right)=\frac{\frac{f\left(R\right)}{attempts_{\left(R\right)}}}{\sum_{i\in V}f\left(i\right)},$$

where *f*(*R*) is the count of failure, *attempts_(R)_* the number of attempts made to connect *R* with other nodes, and $\underset{i\in V}{\sum }f\left(i\right)$ the total failure ratio while connecting all the nodes.

The PRM has a fast sampling rate and generates the shortest path within a short time. Two time-consuming phases of the PRM are during free sample generation and during the test, where the local method can yield a path between the new sample and the configuration in the graph. If *T*_1_ and *T*_2_ denote the tentative time required in two corresponding phases, then the total time required by the algorithm can be calculated as follows

(6) $$T_{TOT}=\omega \left(R\right)\times n\times \left(T1+T2\right),\;\;T_{1}\ll T_{2}.$$

### 3.2. ABC

Compared with other algorithms, the ABC algorithm assists in global and local search in each iteration. Consequently, the probability of obtaining the optimal parameters is improved significantly, and hence local optima are avoided. The ABC algorithm is inspired by the intelligent foraging behavior of honey bees. The ABC algorithm contains three types of bee [28,42], i.e., employed, onlooker, and scout bees. The employed bees search for food around the food source in their memory; additionally, they share information regarding the food sources to the onlooker bees, which tend to select good food sources from those discovered by the employed bees. The scout bees are transformed from a few employed bees, which circumvent their food sources and search for a new one. 

The general structure of the ABC algorithm is briefly described below.

#### 3.2.1. Initialization Phase 

Let $x_{i}\left\{i\in 1,\ldots ,\;N_{s}\right\}$ represent a feasible solution to the optimization. Each solution $x_{i}$ contains *S* parameters to be optimized $\left\{x_{i,j},j=1,\ldots ,S\right\}$. Hence, we can initialize the food positions, i.e., the solution, as follows

(7) $$x_{i,j}=l_{j}+rand\;\left(0,1\right)\left(h_{j}-l_{j}\right),$$

where *h_j_* and *l_j_* are the higher and lower limits of the parameter *j*, respectively, and *rand* (0, 1) is a random number between 0 and 1.

#### 3.2.2. Employed Bee Phase

Each employed bee is associated with the food position. The employed bee seeks a new food position *y_i_* using its current food position $x_{i}$ as follows

(8) $$y_{i,j}=x_{i,j}+\emptyset_{i,j}\left(x_{i,j}-x_{r,j}\right)$$

where *r* is a randomly selected candidate solution (*r≠i*), and ∅*_i,j_* a random number within [−1, 1]. Subsequently, the employed bees compute the nectar amount of *y_i_*. If the fitness value of *y_i_* is better than that of *x_i_*, then *x_i_* is replaced with *y_i_*; otherwise, *x_i_* is unchanged.

#### 3.2.3. Onlooker Bee Phase 

An onlooker bee evaluates the food positions based on a roulette wheel selection mechanism, which is expressed as

(9) $$p_{i}=\frac{fit_{i}}{\sum_{j=1}^{SN}fit_{j}}\;,$$

where *fit_i_* is the fitness value of the *i*-th coordinate. Once the onlooker bees select the food positions, they generate new positions and evaluate them as well. 

#### 3.2.4. Scout Bee Phase

If a position cannot be improved over a predefined number of cycles, then the food source is abandoned and replaced by a random food position (7). 

### 3.3. Problem Formulation

We consider a UAV scenario as a mobile data collector to gather information from a set of *N* sensors, denoted as $\left\{s_{n},1\leq n\leq N\right\}$. The position of the sensor *S_n_* is expressed as$\;p_{n}\in R^{2\times 1}$. Clustering sensor nodes is crucial to lessen the network delay and avoid collision during the data collection period. Therefore, sensor nodes are clustered using the unequal clustering algorithm, as discussed in [43,44]. At first, sensor nodes share their head status (i.e., node identifier, residual energy, and location coordinates) to all the sensor nodes within their range. The node with the highest energy is chosen as CH for that region. If any node is selected as CH, no other CH will be selected within its competition range. After a CH is nominated, sensor nodes send a join request to the CH. Then, the CH selects its cluster members based on distance and the number of cluster members it can accommodate. CHs are accountable for gathering data from their cluster members before UAV arrives. At every round, after UAV-to-CH communication, the remaining energy of CH is compared with the minimum energy required to be CH. If the remaining energy of a CH is lower than the minimum required energy, the CH selection process is restarted.

Each sensor senses the surrounding environment and transfers information bits to the CH; subsequently, the CH aggregates and uploads data to the UAV using a hybrid medium access control (MAC), as discussed in [44]. CHs use line-of-sight (LOS) communication to transfer data to UAV. Intelligent computing methods can be considered to minimize the computation time as well as the energy of power-constrained devices. Different cost-optimization strategies are discussed in [45] to minimize power-constrained devices’ computation time and energy. We will consider one of the efficient computing techniques in our upcoming project. The UAV flies at a certain height, *H*, and velocity, *v*, from the start point (*X_s_, Y_s_, Z_s_*) to the endpoint (*X_e_, Y_e_, Z_e_*). We assume that $\left|\left|X_{s}-X_{e}\right|\right|\leq v_{max}\times T$, such that there exists at least one feasible trajectory from *P_start_* to *P_end_* within *T* even when the velocity is at the maximum. The UAV’s starting and final positions are assumed to be predetermined, with horizontal coordinates denoted as$\;X_{s},\;X_{e}\in R^{2\times 1}$, respectively. The UAV’s trajectory projected on the ground is denoted as$\;X_{\left(t\right)}\in R^{2\times 1},\;0\leq t\leq T.$ *T* is partitioned into m time slots for convenience, i.e., T=mδt, where δt denotes the elemental slot length for the ground sensors to approximate the UAV’s location.

**Figure 2:**
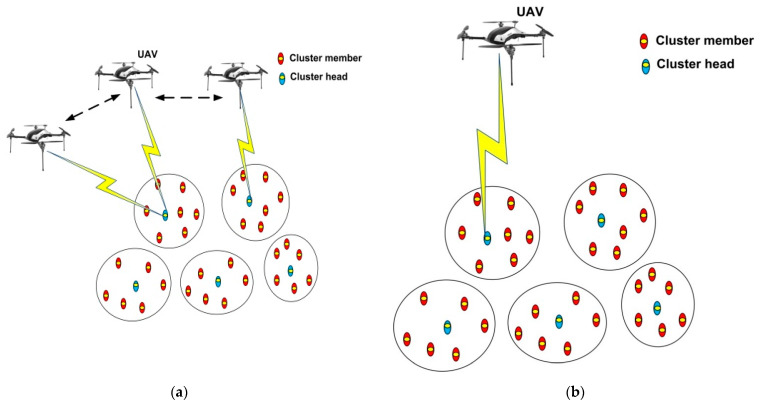
Data collection models in UWSNs: (**a**) data gathering while flying and (**b**) data gathering while hovering.

We assume that the sleep and wake-up mechanism is employed, and one CH, at most, wakes up to communicate with the UAV at each time slot. We denote the wake-up schedule variable as *x_n_[m]*, where *x_n_[m]* = 1 if *CH_n_* wakes up at time slot *m*, and *x_n_ [m]* = 0 otherwise. Hence, we have $\overset{N}{\underset{n=1}{\sum }}xn\left[m\right]\leq 1,\;\forall m$. If *x_n_[m]* = 1, subsequently, $s_{n}$ transmits data with transmission energy *E_trans_*. During no transmission, the UAV flies at the maximum speed *v_max_* to minimize the total flight time. The UAV receives data when it is flying or hovering [46]. Both the cases considered for data collection are shown in Figure 2 and modeled as follows.

#### 3.3.1. Data Gathering while Flying

In the majority of the flight time, the UAV gathers data while it is flying. During this time, $0>v\geq v_{max}$, the UAV collects data from the ground nodes. The flight time of the UAV is determined as $F_{\left(t\right)}=(y_{n}-x_{n}/v$). The sensors’ transmission distance might change slightly when the UAV is flying; therefore, the UAV’s height and speed should be adapted to achieve better network performances. The LOS ground-to-air channel model between the UAV and sensors with path loss exponent ∝≥ 2 was adopted. In this model, the prompt data rate at time *t* is expressed as

(10) $$R\left(t\right)=\frac{1}{2}W\log2\left(1+\frac{\beta }{{\left(v{\left(t\right)}^{2}+H^{2}\right)}^{\frac{\alpha }{2}}}\times E_{trans}\right),$$

where $t\in \left[0,f_{\left(t\right)}\right]$; *W* is the bandwidth, β is the reference signal-to-noise ratio at the reference distance of 1 m; *H* is the height of the UAV; $E_{trans}$ is the transmission energy, which satisfies the total energy constraint

(11) $$\int_{i=0}^{F_{\left(t\right)}}E_{trans\left(i\right)}dt\leq E_{T}.,$$

#### 3.3.2. Data Gathering while Hovering

If the SNs are likely to be outside the UAV’s connectivity owing to the high speed, then the UAV hovers with *v* = 0 for a specific time. Therefore, the sensors can upload the remaining data to the UAV during the hovering time, thereby minimizing packet loss significantly. The time spent hovering above the location is concise and is expressed as *H(t*) = $t_{h,n}\left(x_{n},\;y_{n},z_{n}\right)$. As the UAV is static at this point, the CH’s energy is simplified to the following

(12) $$t_{h,n}\left(x_{n},\;y_{n},z_{n}\right)\times E_{trans}\leq E_{T}.$$

## 4. Hybrid Algorithm for UAV Path Planning

In this section, the proposed HPP algorithm is presented in detail. After summarizing our network model, we present the constraints of dynamic environments for optimization and the proposed algorithm.

### 4.1. Network Model

The proposed hybrid path planning (HPP) algorithm comprises classical PRM and ABC algorithms. The PRM calculates the shortest path in collision-free environments but cannot adapt according to network constraints. Therefore, we considered a modified ABC that obtains optimal solutions even in harsh environments using optimization methods. The path coordinates from the hybrid solution should be free from static and dynamic obstacles. It should not pass through interdicted areas such as military offices and airports, avoid all possible threats such as radar networks, and ensure that the UAV encompasses the WSN collection zones well. Minimization of flight time and energy is necessary for UWSN-based emergency environments. In our study, the SNs are static and deployed randomly. Moreover, the SNs are clustered, and UAV and SNs are equipped with GPS for achieving higher efficiency of location and timing information. We assume a quadcopter UAV for our work so that the movement and speed of the UAV can be optimized concerning the environmental factors. As a result, the UAV is capable of hovering in the target area.

The UAV incorporates two transceivers, one of which is used to broadcast beacon signals and receive registration packets. In contrast, another is used to transmit scheduling information and receive data packets. The UAV is equipped with a directional antenna with flare angle *φ* = 60° because directional communication offers many benefits such as extended transmission, minimized delay, and spatial reuse. The sensors lying on the coverage area and receiving beacon signals from the UAV become active. If a sensor does not receive signals or the received signal strength is weak, it goes to sleep mode to save energy. In this way, the directional antenna limits the UAV coverage and overcomes the directional deafness problem. The UAV moves in a 3D environment with an absolute velocity that optimized the UAV flight time reduction. Different constraints of dynamic and complex environments considered for optimizing the proposed path are discussed in the following subsection.

### 4.2. Constraints for Path Planning

#### 4.2.1. Collision-Free Path

A collision can occur in either static or dynamic obstacles. To achieve a feasible path plan, the characteristics of all obstacles must be identified and avoided. Hence, if the function *g*(*X_i_, Y_i_*) returns the land’s altitude at a certain point, then the equation that expresses the total number of waypoints falling into an obstacle is as follows.

(13) $$W_{obs}=\sum_{i}Z_{i}A_{i}=\{\begin{array}{c} 1\;if\;Z_{i\;}\neq g\left(X_{i},\;Y_{i}\right)\\ 0\;otherwise. \end{array}\;,$$

#### 4.2.2. Threat Modeling

The UAV may encounter specific threats during its flight; those threats must be detected and removed. If {*X_t_, Yt, Z_t_*} are the coordinates of the threat and *R_threat_* is the range of the threat, then the exposure of the UAV at {*X_i_, Y_i_, Z_i_*} in the area of threat is expressed as

(14) $$Threat_{in}\left(i\right)=\{\begin{array}{c} \frac{R^{2}t}{(d^{2}{}_{XOY\;}-Z^{2}{}_{i\;})Z_{i\;}}\;if\sqrt{{\left(X_{i\;}-X_{t\;}\right)}^{2}+{\left(Y_{i\;}-Y_{t\;}\right)}^{2}}<R_{t\;}\\ 0\;\;\;\;\;\;\;\;\;\;\;\;\;\;\;\;\;\;\;\;\;\;\;\;\;\;\;\;\;\;\;\;\;\;\;\;\;\;\;\;\;\;\;\;\;\;\;\;\;\;\;\;\;\;\;otherwise \end{array}\;$$

where $d_{XOY}=\sqrt{{\left(X_{i\;}-X_{t\;}\right)}^{2}+{\left(Y_{i\;}-Y_{t\;}\right)}^{2}+Z^{2}i}$ is the Euclidean distance of the UAV at the *i*th waypoint and threat.

#### 4.2.3. Relative UAV-WSN Height

The height of the UAV’s flight path affects the performance of the UWSNs. A higher altitude results in a more considerable transmission distance for the SNs and higher energy consumption. The proposed algorithm attempts to minimize the elevation of the UAV, which is calculated as follows

(15) $$Alt_{\left(j\right)}=\{\begin{array}{c} \frac{\sum_{j=1}^{W_{p}}\left(Z_{j\;}-g\left(X_{j\;},\;Y_{j\;}\right)-Min_{h}\right)}{W_{p}}Z_{j\;}>g\left(X_{j\;},\;Y_{j\;}\right)+Min_{h}\\ \frac{\sum_{j=1}^{W_{p}}\left(g\left(X_{j\;},\;Y_{j\;}\right)+Min_{h}-Z_{j\;}\right)}{W_{p}}\;\;\;\;\;\;\;\;\;\;\;\;\;\;\;\;\;\;\;\;\;\;\;Otherwise \end{array}$$

where *Min_h_* is the minimum desired height of the UAV, *W_p_* the total number of waypoints, *g (X_j_, Y_j_*) is the terrain height at point (*X_j_*, *Y_j_*), and (*X_j_, Y_j,_ Z_j_*) the *j*th waypoint of the UAV.

#### 4.2.4. Turning Angle

A smaller turning angle yields a smoother path. Hence, for a UAV at the *j*th waypoint, the least desired turning angle can be calculated as follows

(16) $$\theta_{j}=\left|tan^{-1}\frac{Y_{\left(j+1\right)\;}-Y_{j\;}}{X_{\left(j+1\right)\;}-X_{j\;}}\right|.$$

Subsequently, the effect of the turning angle on the complete path is expressed as

(17) $$Effect{\;}_{turn}=\overset{W_{p}}{\underset{i=0}{\sum }}\theta_{i}.$$

#### 4.2.5. UAV Speed Optimization

The UWSN applications’ goal is to include all the CHs for the network’s fairness and remain sufficiently inside their communication range to receive data efficiently. We assume that the UAV maintains the least possible altitude during the data-gathering phase. If *r* is the coverage radius of the UAV on the ground flying with speed *v*, then the optimization of the UAV’s speed for minimizing the flight time and maximizing data collection is expressed as

(18) $$Max\left(speed\right)=v_{max}$$

(19) $$v_{\left(k+1\right)}=v_{\left(k\right)}+t_{i}v_{w}+\rho \Delta t$$

(20) $$\mathrm{Subject}\;\mathrm{to}:v\leq 2\frac{\sqrt{r^{2}\_UAV\_CH_{i}\_distance^{2}}}{t_{i}},$$

where *t_i_* is the UAV-to-CHi communication time, *v_w_* the wind speed, *ρ* the density of air, ∆*t* the changed instance of time, and *v_(k)_* the UAV’s speed in its previous position.

#### 4.2.6. Flight Time Coordination

Time is critical in UAV path planning. Flight time can be considered as the time the UAV spends in sensor fields to obtain sensed information or the time required to fly to the target area and return to the BS. Assuming that *D_size_ci_* is the size of data in a cluster, the time required for any CH to transmit its data is expressed as

(21) $$t_{i}=\frac{D\_size_{c_{i}}}{data\_rate}\times UAV\_CH_{i}\_distance.$$

If (*x_ci_,y_ci_*) is the coordinate of the *i*th CH and (*x_u_(t), y_u_(t),z_u_(t)*) is the position coordinates of the UAV at time instant *t*, then the shortest *UAV_CH_i__distance* is expressed as

(22) $$UAV\_CH_{i}\_distance=\sqrt{{\left(x_{u}\left(t\right)-x_{ci}\right)}^{2}+{\left(y_{u}\left(t\right)-y_{ci}\right)}^{2}+{\left(z_{u}\left(t\right)-0\right)}^{2}}$$

(23) $$T_{Total}=T_{Fly}+t_{i\left(total\right)},$$

where *t_i_(total*) is the *UAV_CH* communication time expressed as $\overset{n}{\underset{i=1}{\sum }}t_{i}$, $s\in \left[s_{min},s_{max}\right]$ is the range of speed, and *L_i_* is the path length at the *i*th waypoint. Hence,

(24) $$T_{Fly}=\sum_{i=1}^{W_{p}}T_{Fly\left(i\right),}$$

where

(25) $$T_{Fly\left(i\right)}=\left[\frac{L_{i}}{s_{min}},\frac{L_{i}}{s_{max}}\right].$$

### 4.3. Proposed Hybrid Path Planning

The pseudocode of the proposed HPP algorithm is provided in Algorithms 1 and 2. Algorithm 1 uses the PRM to create a roadmap of the UAV’s flight path. First, the starting and goal points of the UWSN are determined. From this *P_start_* position, neighboring path coordinates are randomly selected and listed in *R*. Subsequently, coordinates that do not collide with the starting point are listed in *V*. Path coordinates in *V* are sorted by the distance on the road to the destination. Nodes that lead the UAVs closer to the destination and are free from obstacles are selected and connected to generate a simple path, which is also the shortest. The process is repeated based on the number of iterations to obtain the complete path from *P_start_* to *P_end_*.
**Algorithm 1.** Roadmap construction based on PRM**Input:** *P_start_* = starting position coordinates (*X_s_,Y_s_,Z_s_*) *P_end_* = end-position coordinates. (*X_e_, Y_e_, Z_e_*) *Iter_max_* = number of nodes to complete the roadmap **Output:** Shortest and collision-free path /*Initialize network*/ 1: **begin** 2: *V*←∅ 3: *E*←∅ 4:  i=0; 5:    **for**$(i\leq Iter_{max}$) **do** 6:             **if** (*R*← random configuration from *P_start_* in free space) && (*R*← collision-free), **then** 7:         *V*← *V*
$\cup^{\;}$
*R* 8:             **end if** 9:             **repeat;** 10:    **end for** 11:  **for** all *R*
∈
V, **do** 12:               $N_{P_{start}}$← closest neighbors of *R* from *V* sorted by increasing distance toward *P_end_* //finds the coordinates for the shortest path 13:         **for** all $R^{\prime }\in$
$N_{P_{start}}\;$**do** 14:             **if** not connected ($R,R^{\prime }$) && not obstacle ($R-R^{\prime }$) **then** //if the points are not connected, and the local planner finds a path between points 15:                   *E*←*E*
$\cup^{\;}$ ($R,R^{\prime }$) 16:             **end if** 17:         **end for** 18:   **end for** 19:   return the generated path 20: **end**
**Algorithm 2.** HPP algorithm**Input:** *P_start_* = starting position coordinates (*X_s_,Y_s_,Z_s_*) *P_end_* = end-position coordinates. (*X_e_,Y_e_,Z_e_*) *Iter_max_* = number of nodes to complete the roadmap Roadmap constructed by Algorithm 1 **Output:** The optimized path from the point of origin (*X_s_, Y_s_, Z_s_*) to the point of destination (*X_e_, Y_e_, Z_e_*) /* initializes the network*/ 1: **begin** 2:  **for**
$\left(i=1\;to\;Iter_{max}\right)\;$**do** 3:        **for** each employed bee, **do** 4:              Use the food position, i.e., path coordinates generated in **Algorithm 1** 5:        **end for** 6:        **for** each onlooker bee, **do** 7:    Select new food sources based on the probability values 8:              Compute the collision and threat probability using (13) and (14) 9:                **if** the collision is detected as in (13) && threats as in (14), **then** 10:                         Avoid that coordinates 11:                         **repeat** step 10–13; 12:               **else** 13:                       Compute Optimized values of UAV’s speed //to minimize the flight time using the highest possible speed 14:                       Compute the turning angles with minimum effect for a smooth path from (17) //smoothness in path minimizes the energy consumption and flight time of the UAV 15:                     Generate a new food source with optimized parameters 16:               **end if** 17:              **while** UAV is in the region of sensor nodes, **do** 18:                     Compute least possible height from sensors as in (15) //to minimize the energy of the sensor by reducing the transmission distance 19:        Compute the optimized value of UAV’s flight time for time-efficient data collection as in (24) //to increase the UAV-to-sensor communication time, optimize the speed 20:              **end while** 21:             Show the new path coordinates ($X_{new},\;Y_{new},\;Z_{new})$ 22:        **end for** 23:        **for** each scout bee, **do** 24:              Evaluate the fitness of the coordinates used by the onlooker bee 25:     Evaluate the fitness of the coordinates used by the employer bee 26:            **if** the coordinates generated by the onlooker bee is better than those of the employer bee, **then** 27:                 Replace the coordinates of the employer bee with a new solution 28:            **else** 29:                  Use the coordinates generated by the roadmap (Algorithm 1) 30:            **end if** 31:        **end for** 32:      Memorize the best path 33: **end for** 34: **end**

Initially, the UAV follows the roadmap generated by Algorithm 1. The generated path is optimized by considering constraints such as dynamic obstacles and other issues about the complex environment, as discussed in the subsection above. The ABC algorithm imitates the behavior of biological bees. The employed bees are supposed to follow the roadmap, whereas the onlooker bees continue to seek new coordinates and calculate their collision and threat probability using (13) and (14). If collision or any other threats are detected, then the onlooker bees search for new food sources and execute the steps above. If the coordinate is free of any threats and collision, the optimized values of speed, height, and turning angles are computed, as in (15), (17), and (18), to minimize the total flight time and the network energy. The HPP algorithm is shown in Algorithm 2.

**Figure 3:**
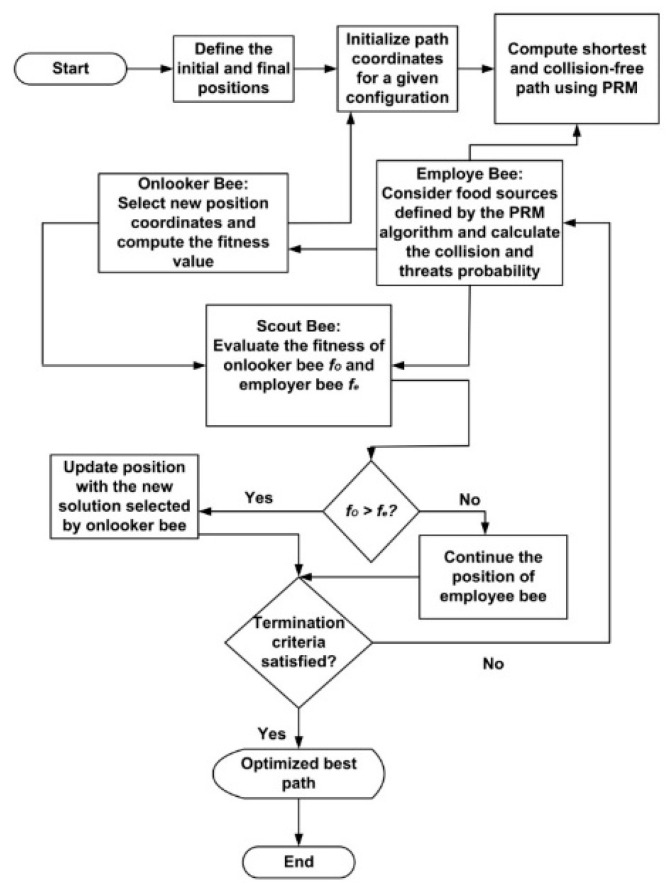
Operation flow of the proposed HPP algorithm.

Figure 3 shows a comprehensive operation flow of the proposed hybrid algorithm. First, the starting and goal points of the application area are initialized. The PRM is used to calculate the shortest and collision-free path from the start point to the goal point. Initially, the shortest path determined by the PRM algorithm is used. However, the ABC algorithm is used to optimize the path coordinates for various environmental constraints. The ABC algorithm uses three different types of bees. In our proposed method, the employer bees follow the path generated by the PRM. The onlooker bees seek new path coordinates and calculate the fitness probabilities for new coordinates. The scout bees compare the fitness value of the onlooker bee to that of the employer bee. If the new coordinate’s fitness value is higher than the roadmap’s coordinates, new coordinates are selected considering the time and energy minimization of the proposed UWSN; otherwise, the new coordinates are discarded. Hence, static roadmap generation and dynamic path planning are involved in obtaining the shortest and safest paths for emergency environments. 

## 5. Performance Evaluation

In this section, the proposed HPP algorithm’s performance is evaluated and compared with conventional PRM and ABC algorithms. In our performance study, all the path planning algorithms under evaluation are simulated using the MATLAB R2020a simulator.

### 5.1. Simulation Environment

In our simulation, SNs are randomly distributed in an area measuring 500 m × 500 m. Among the total SNs deployed, 10% of them are selected as CHs responsible for transmitting data to the UAV. We investigated an efficient MAC protocol for UAV-to-CH communication in our previous study [35], and this is used in the current study. The UAV uses a hybrid MAC protocol for UAV-to-CH communication. The UAV has a roadmap of its flight path, provided by the PRM algorithm before flying. The map provided by the PRM is the shortest and free of static and dynamic obstacles, and threats from prohibited areas. It follows the shortest path with a predefined height and speed until it encounters the complex environment’s obstacle and threat. In this case, the UAV uses the ABC to avoid threats and seeks the shortest, safest, and the smoothest path to accomplish its mission. For an efficient operation of the protocol, we optimized the UAV’s height and velocity such that every CH has access to the UAV at least once during the flight time.

We have considered some key metrics to evaluate the proposed MAC’s performance and compared it with existing protocols. The performance metrics used for the evaluation are as follows.

#### 5.1.1. Flight Time for Flight Path

As our study targets disastrous and emergency environments, the timely and reliable environmental information transfer is emphasized. The time for UAV-to-CH communication is limited owing to the high mobility of the UAV. Hence, reducing the UAV-to-CH communication time affects the network performance and is not acceptable. Therefore, in our study, we optimized the UAV velocity during data and non-data collection periods to minimize the total flight time without degrading the network performance. The UAV uses the maximum speed when it does not communicate with the SNs, and the value changes adaptively during the communication. The total flight time of the network is expressed as

(26) $$F_{Tot}=flying\;time+hovering\;time+UAV-to-CH\;communication\;time$$

#### 5.1.2. Network Lifetime

The operation of the UWSN is subjected to several constraints, among which, available energy of the nodes is one of the most critical. Furthermore, network lifetime is an essential parameter in UWSNs as it represents the number of dead nodes generated in every simulation round because of energy exhaustion. In UWSNs, both UAVs and WSNs are power-constrained devices operated by batteries. Specifically, the SNs cannot be charged and replaced repeatedly. Hence, the energy of the SNs determines the lifetime of the UWSNs. The primary goal of our study is to minimize the energy consumption of the SNs. However, we have also attempted to use the UAVs’ minimum possible energy to accomplish the defined mission. To minimize the energy of the SNs, we minimized the period for which the CHs remained active by synchronizing the UAV’s flight time with their wake-up cycles. Besides this, we used the clustering approach, which enabled the extension of the network lifetime. To minimize the UAV energy, we have generated the smoothest and shortest path coordinates.

#### 5.1.3. Convergence Time

Convergence time is the time required by the proposed hybrid algorithm to compute the number of iterations and decide the best coordinates to move to. Therefore, the convergence time from one stage to another is calculated and compared with the literature’s widely discussed algorithms. In our proposed algorithm, several iterations are involved for both PRM and ABC algorithms. The roadmap is generated beforehand using the PRM algorithm, and thus the convergence time of PRM does not impact the flight time of the UAV. Because the proposed UWSN model’s performance depends on the convergence time of the ABC algorithm, the convergence time of ABC is analyzed and compared with other path planning algorithms in the following section.

#### 5.1.4. Flight Path Encompassed

The UAV must visit all the nodes and collect data from them during its flight time. Hence, the flight path must encompass all the CH locations in the WSN deployment region. We performed optimization to minimize the UAV path. The UAV’s distance in the proposed UWSN application during its mission is calculated and then compared with other algorithms’ results. The path encompassed by the UAV during its mission is calculated in meters.

#### 5.1.5. Packet Delivery Ratio

The packet delivery ratio is the ratio of successfully delivered packets among the transferred packets. It is expressed as

(27) $$Packet\;delivery\;ratio=\frac{\sum^{\;}Number\;of\;received\;packet}{\sum^{\;}\;number\;of\;sent\;packets}$$

Having the maximum number of packets delivered to the UAV is highly desirable to achieve better network performances. In our proposed algorithm, we considered data collection while the UAV is hovering to minimize data loss due to the limited contact time of the UAV and CHs. 

#### 5.1.6. Computational Complexity

The proposed algorithm’s computational complexity is calculated as the time required by the algorithms to find the best possible path coordinates towards the target area. Finding an accurate running time of the algorithm concerning its input size is challenging. The asymptotic time complexity of the algorithm is projected based on the instructions provided in the algorithm. In the proposed algorithm, PRM and ABC are used. The time complexity of the PRM algorithm is given by the *O (n log n*) where *n* is the total number of iterations. The HPP algorithm’s performance depends on the time complexity of the ABC algorithm to generate trajectory points in the dynamic environment. Hence, the proposed HPP’s time complexity is O (*nt*), where *t* is the number of threats.

The simulation parameters used in our performance study are listed in Table 3.

### 5.2. Simulation Results and Discussion

In this section, the simulation results are summarized and compared with those of the conventional PRM and ABC algorithms. The simulation is performed in a 3D scenario. The UAV uses two transceivers operating concurrently. Our primary aim is to reduce the network delay by the minimal usage of the sensor’s energy to enhance network performance. In the simulation, the UAV receives information regarding the CHs and their locations while it is moving. The UAV’s path encompasses all the sensors while it moves within the WSN area. The UAV collects data from the CHs while it is moving or hovering to reduce packet loss. We have optimized the height, speed, threats, and flight time to enhance the proposed system’s performance.

**Figure 4:**
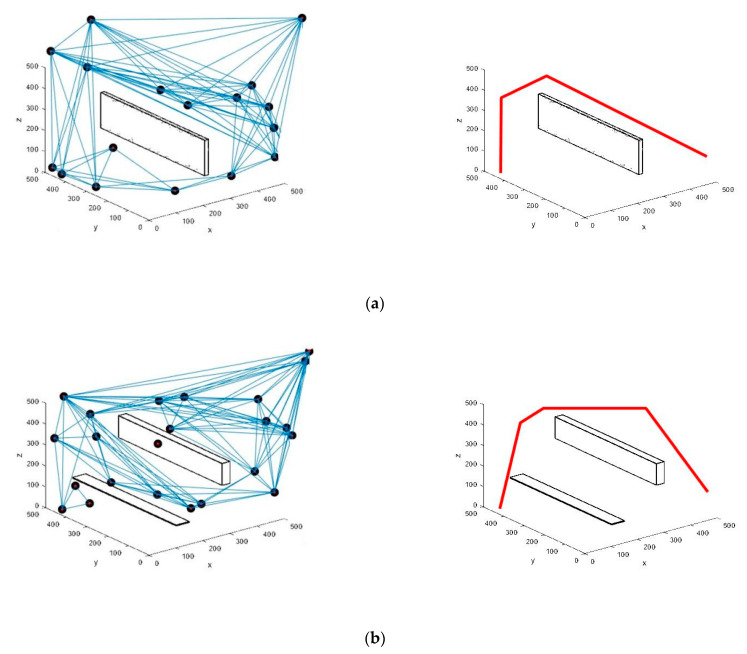
UAV’s path using PRM algorithm in two different scenarios: (**a**) single static obstacle and (**b**) multiple obstacles.

The scenarios and results of the PRM implementation are shown in Figure 4. In Figure 4a, PRM is implemented with a single static obstacle. The shortest collision-free roadmap designed by PRM is also presented aside. Likewise, in Figure 4b, the simulation is done in the scenario with multiple obstacles, and the shortest path is designed.

**Figure 5:**
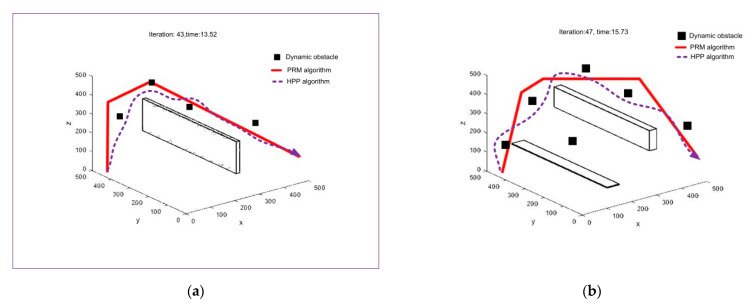
UAV’s path using the proposed HPP algorithm with static and dynamic obstacles: (**a**) with single static obstacle and four dynamic obstacles and (**b**) multiple static obstacles and six dynamic obstacles.

The roadmap acquired through the PRM algorithm in Figure 4 is used as the proposed HPP input. Our simulation is carried out with a varying number of static and dynamic obstacles, and the obtained results are shown in Figure 5. In Figure 5a, we use a static obstacle and four dynamic obstacles. The proposed algorithm finds the way to the target area, avoiding all the obstacles. In Figure 5b, two static obstacles and six dynamic obstacles are used. We consider dynamic obstacles of the same shape and size, which are randomly generated during the simulation. With the increase in the number of static and dynamic obstacles, we can notice that both the number of iterations required and the time taken to reach the goal point increase to some extent. Thus, our proposed algorithm performs well to find the shortest and collision-free path in a dynamic environment with sudden threats and obstacles.

**Figure 6:**
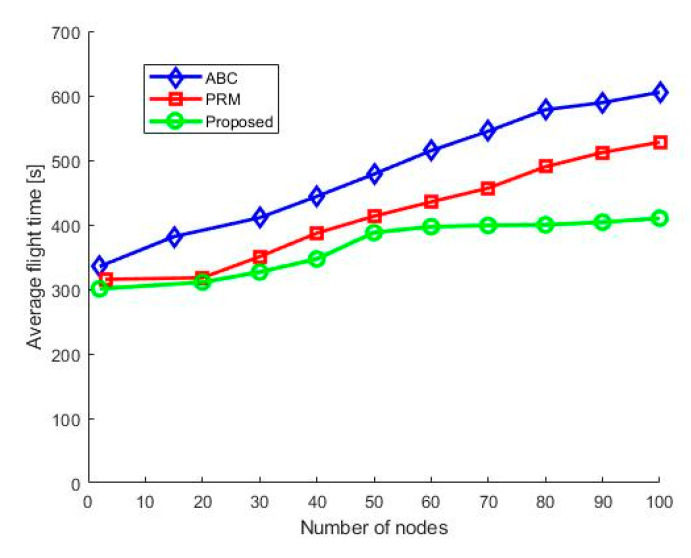
Average flight time of UAV in the network.

As shown in Figure 6, the average time required by the UAV to encompass the assigned path is calculated. For the simulation, we have focused on the UAV’s time to traverse the WSN deployed area. We have also compared our results with those obtained using the classical PRM and ABC schemes. The results validate that the proposed hybrid method optimizes the UAV’s total flight time for a specified simulation time and condition. The reduction in flight time is due to the optimized UAV height and speed and the proposed algorithm’s high convergence rate.

**Figure 7:**
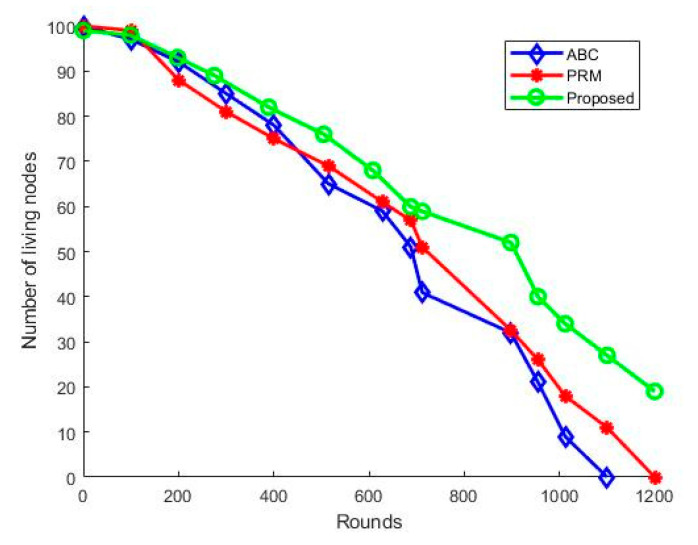
Network lifetime.

The lifetime of the proposed UWSN is calculated and analyzed for all rounds. The simulation is performed for the specified number of rounds to verify the number of alive nodes. We have considered hybrid MAC and clustering for all three algorithms. The number of live nodes is increased for all three algorithms compared to the conventional PRM and ABC algorithm due to the hybrid MAC and clustering. Figure 7 shows the lifetime of the three path planning algorithms. It is apparent that the number of live nodes is proportionally low for the ABC and PRM schemes. However, for the proposed hybrid scheme, the number of living nodes is higher. This is due to the optimized hybrid path plan algorithm. Furthermore, the CH wake-up schedule is synchronized with the UAV flight time. Using this mechanism, the CH need not remain active for a longer duration, reducing energy consumption. This significantly affects the energy efficiency of the network. 

**Figure 8:**
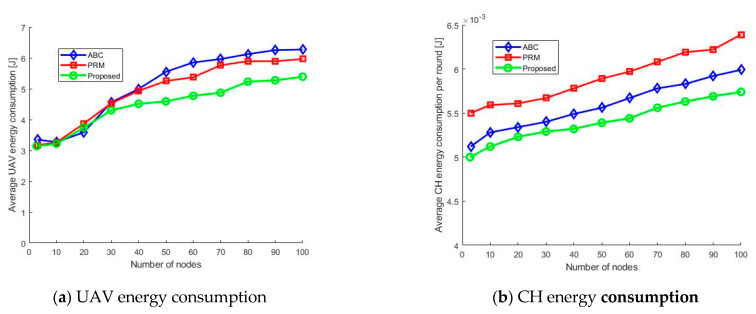
Energy consumption.

The WSNs and UAVs’ energy consumption remains a design concern, as a reasonably long network lifetime is desirable. The average energy consumption of the UAV and SNs are calculated and analyzed, as shown in Figure 8. We have compared the obtained results using the energy consumption of the PRM and ABC algorithms. From the results shown in Figure 8a, it is apparent that the energy usage of the UAV decreased when using the proposed hybrid path planning method. We have focused on the energy consumption of the UAV during its flight and communication with the SNs. Minimization in energy consumption is possible owing to the optimization process and reduced flight time, as shown in Figure 6.

As shown in Figure 8b, we calculated the average energy required by any CH to communicate with the UAV. We ran the simulation for the specified number of rounds and then calculated the average time of CHs per round. From the figure, it is evident that the energy consumption of the CHs in the proposed algorithm decreased slightly in every round of UAV-to-CH communication. This significantly helps to reduce the energy consumption of the CHs when it is operated for a more significant number of rounds. Consequently, the lifetime of the overall network can be improved.

**Figure 9:**
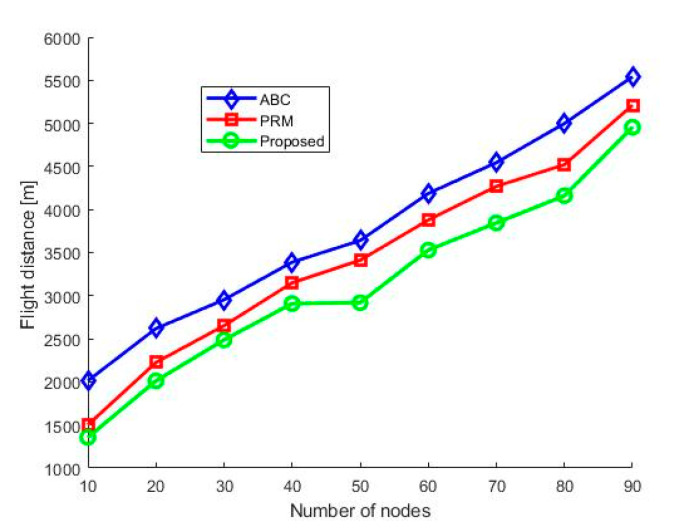
Total distance encompassed.

Figure 9 shows the total distance traveled by the UAV to encompass the sensor deployed region. The results show that the proposed method’s distance is minimal for the three simulated methods. The reduction in flight path is because the HPP uses the shortest path provided by the PRM unless it encounters a collision and obtains an optimized path provided by the ABC to manage the complex environment’s different constraints. Initially, the PRM path planning performs well; however, it cannot handle the dynamic obstacles, and the height and speed are not optimized. We aim to minimize the flight distance to complete the mission quickly to lessen the damage in emergencies.

**Figure 10:**
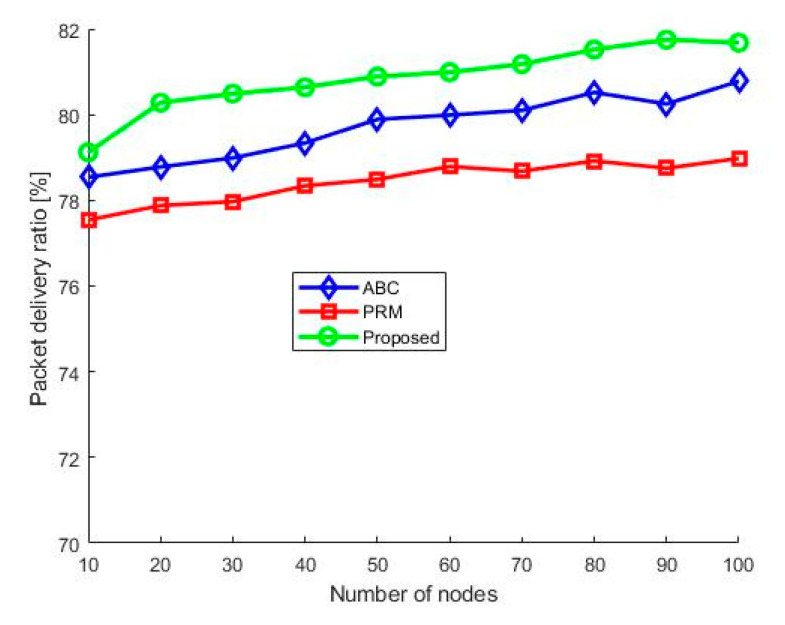
Packet delivery ratio.

Figure 10 shows the PDRs of the PRM, ABC, and the proposed algorithm. According to the results, the proposed algorithm’s PDR remains higher than 80% after the UAV entered the WSN region. The change in packet loss from the proposed method is minimal due to the speed and height adaptation during the data collection time. Additionally, UAV hovers when data collection is not completed. The proposed method is then compared with the PRM and ABC algorithms. From the comparison, we can conclude that the number of packets received from the proposed algorithm is higher than those from the PRM and ABC algorithms. The PRM shows the lowest packet delivery rate because it does not function appropriately in complex environments and adapt in accordance with the constraints.

**Figure 11:**
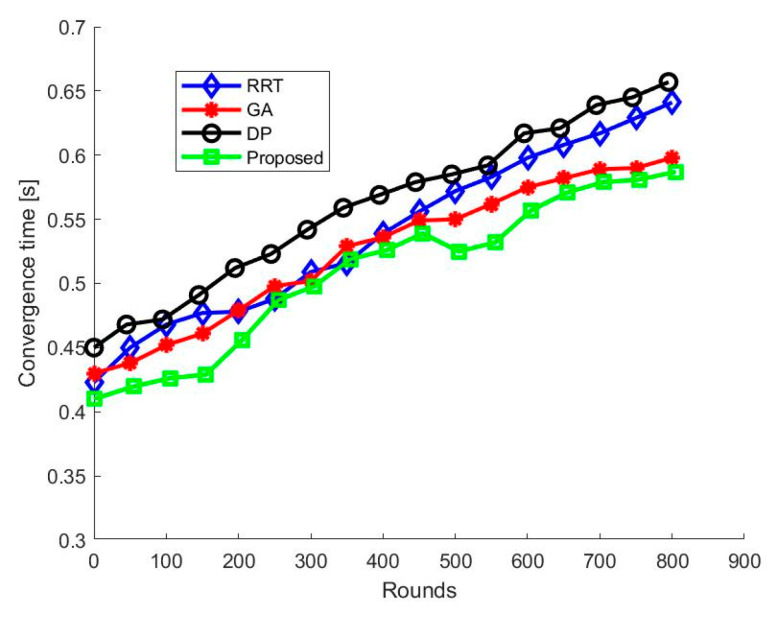
Convergence time.

We used the optimized ABC algorithm for the local path plan because of its fast convergence capacity. We evaluated the convergence time of the ABC algorithm and compared it with other widely used path planning algorithms (i.e., RRT, GA, and DP) in Figure 11. We used the same network configuration for all the algorithms used in the comparison. Based on the obtained results, it is apparent that the convergence time of HPP is the shortest for almost all the rounds. In our proposed HPP algorithm, the classical ABC algorithm is modified by optimizing various constraints. As a result, the proposed algorithm offers a faster convergence speed than the other three popular algorithms.

## 6. Conclusions and Future Works

A novel path planning algorithm that applies UAVs as an efficient data collector from WSNs in emergency environments is proposed herein. Considering the advantage of the PRM algorithm in obtaining the shortest collision-free map and that of the ABC in operating and converging quickly, we have addressed the issues of slow convergence and easy entrapment in the locally optimal solutions observed in other algorithms. The proposed HPP algorithm has an optimization process for different flying parameters such as altitude, path length, flying time, and hovering time. It can effectively obtain the shortest and safest path, as well as managing different dynamic environment constraints. The proposed algorithm performs better than the conventional ABC and PRM algorithms and requires a shorter convergence time. The path coordinates that are obtained through the HPP algorithm can be effectively used for real-world applications. The simulation results demonstrate that the proposed HPP reduces flight time and energy consumption in UWSNs. The HPP algorithm manages fixed and dynamic obstacles, avoids restricted areas effectively, and shortens its planning time. Therefore, it can also be used for real-time path planning.

However, our current work has some limitations. We can consider more realistic and complex simulation scenarios and varying sizes and speeds of dynamic obstacles. Additionally, we can use intelligent computing methods to lessen the computation time, as discussed in the problem formulation. Using a swarm of UAVs with better coordination among UAVs can help improve network coverage in large UWSNs. The precise synchronization of sleep and wake-up schedules with UAV’s arrival time is quite challenging during actual implementation. We can work towards a better synchronization to reduce energy and time in power-constrained sensor devices. We will exploit these in our future work. We will also consider some advanced methods such as mobile edge computing and software-defined network techniques in our upcoming work, because they help minimize the computation costs of delay-sensitive networks.

## Figures and Tables

**Table 1:** Summary of the existing works.

Existing Work	Main Idea	Limitation
[32]	ACO-based path determination	Not suitable for dynamic and emergency environment
[33]	RRT and RRT* planning exploration	Not suitable for 3D dynamic and emergency environment
[34]	Spiral decomposition-based path plan	Not suitable for 3D dynamic emergency environment
[35]	Age-optimal trajectory planning	Not suitable for 3D dynamic emergency environment
[36]	PSO-based path planning	Not suitable for 3D dynamic emergency environment
[37]	ACO-based path planning	Not suitable for 3D dynamic emergency environment
[38]	K-means and simulated annealing based path plan	Not suitable for 3D dynamic emergency environment
[39]	DL trained by GA-based path plan	Do not address 3D dynamic and emergency environment.
[40]	DRL and 6G-based path plan	Ignores the obstacles and void areas of dynamic 3D environments
[41]	Genetic algorithm and ACO-based	Offline based algorithm; ignores the aspects of emergency environments
[42]	DP-based path plan	Considers only one-dimensional sensor networks

**Table 2:** List of notations.

Notation	Definition
*P_start_*	Starting position of UAV $\left(X_{s},Y_{s},Z_{s}\right)$
*P_end_*	Ending position of UAV $\left(X_{e},Y_{e},Z_{e}\right)$
*G*	Graph
*V*	UAV’s configuration in free space (vertex)
*E*	Connection between vertices
*γ*	Shortest path between nodes in V
*P_l_*	Path length
$N_{P_{start}}$	Close neighbors of Pstart
*R*	Nearest node from Pstart
*R’*	Nearest node from R
*D_max_*	Maximum distance
*D(R,R’*)	Euclidean distance between R and R’
*w(R*)	Path complexity of R
*f(R*)	Count of failure, ${\mathrm{attempts}}_{\left(R\right)}$
*T*_1_	Time for free sample generation
*T*_2_	Time to obtain a path between nodes in G
$n^{\prime }$	Number of iterations
*N*	Number of sensor nodes
*S*	Optimization parameters
*h_j_*	Higher limit of parameters
*l_j_*	Lower limit of parameters
*r*	Randomly selected candidate solution
*N_s_*	Number of feasible solutions
*p_n_*	Position of sensor nodes
*H*	Height of UAV
*v*	Velocity of UAV
*T*	Flight duration
*M*	Time slots
*E_trans_*	Transmission energy
*F_(t)_*	Flying duration
*H_(t)_*	Hovering time
*E_T_*	Total energy
*W*	Bandwidth
β	Reference signal-to-noise ratio at 1 m
∝	Path loss exponent
*W_p_*	Number of waypoints
*v_w_*	Wind speed
*r*	Coverage radius of UAV
*Min_h_*	Minimum possible height of UAV
ρ	Density of air
$D\_size_{c_{i}}$	Cluster data size

**Table 3:** Simulation parameters.

Parameter	Value
Network simulator	MATLAB
UWSN deployment area	500 m × 500 m
Number of sensor nodes	100
Number of UAV	1
Sensor initial energy	2 J
Packet size (variable)	100–150 bytes
UAV’s height	45 m
Sensors	Static
UAV type	Quadrotor
UAV’s initial energy	5 J
UAV’s initial speed	25 m/s
Antenna type	Directional
Antenna flare angle	60°
Air density	1.225 kg/m^2^
Number of transceivers	2
UAV’s transmission range	200–250 m
Moments of inertia (*I_x_*, *I_y_*, *I_z_*)	1.9757 × 10^−4^ kg/m^2^; 1.9757 × 10^−4^ kg/m^2^; 3.9757 × 10^−4^
